# Confidence-guided outlier refinement and collaborative embedding for unsupervised person re-identification

**DOI:** 10.1038/s41598-026-56072-w

**Published:** 2026-06-19

**Authors:** Jun Zhang, Shuli Cheng, Anyu Du, Haixu Yang

**Affiliations:** School of Computer Science and Technology, Xinjiang University, Ürümqi, 830046 China

**Keywords:** Unsupervised person Re-ID, Contrastive learning, Clustering algorithm, Outlier, Engineering, Mathematics and computing

## Abstract

Unsupervised person re-identification techniques have developed rapidly in recent years. Nonetheless, they still face challenges such as unstable pseudo-label quality and insufficient feature representation, particularly when handling outlier points and complex backgrounds. To address these issues, this paper proposes a joint optimization algorithm of Multi-level Confidence Outlier Refinement (MLCOR) and Collaborative Embedding Method (CEM), which aims to improve the discriminative nature of the embedding space and optimize the accuracy of pseudo-labels. Specifically, Multi-level Confidence Outlier Refinement evaluates the confidence level of outlier points by analyzing the distance relationship between outlier points and their neighboring samples, and classifies them into multiple confidence levels. We design a weighted voting strategy for low-confidence samples to correct the pseudo-labeling of low-confidence points by using the label distribution of neighboring samples, thus reducing noise interference and clustering errors and improving the accuracy of pseudo-labeling. Meanwhile, the Collaborative Embedding Method jointly optimizes global and local features, establishing an effective synergy between global category differentiation and local fine-grained feature learning. By integrating multi-level similarity relationships, this approach not only strengthens the model’s ability to capture subtle differences between samples but also significantly enhances the model’s boundary awareness. Experimental results demonstrate that the proposed method achieves outstanding performance on multiple standard datasets, significantly improving both clustering accuracy and pseudo-label precision, while also exhibiting strong domain generalization and robustness in complex environments.

## Introduction

Person re-identification (Re-ID) is a critical task in computer vision, focusing on recognizing and matching individuals across pedestrian images captured from different cameras and viewpoints^1^. In real-world multi-camera settings, Re-ID facilitates large-scale intelligent surveillance and video analytics by enabling cross-camera association of pedestrian identities. This supports a variety of applications, including intelligent transportation, public safety, retail analytics, and access control. However, deploying Re-ID effectively in practice presents several challenges, particularly due to variations in camera viewpoints, pedestrian appearance diversity, occlusion, and interference from complex backgrounds, most notably the high cost of data annotation. Traditional person re-identification approaches rely on vast amounts of accurately labeled data. However, manual annotation is both time-consuming and costly, and the annotation quality often degrades as the data scale increases. Consequently, unsupervised person re-identification has gained significant attention. In recent years, unsupervised methods have shown promising results, leading to substantial research advancements^2,3^.

**Fig. 1:**
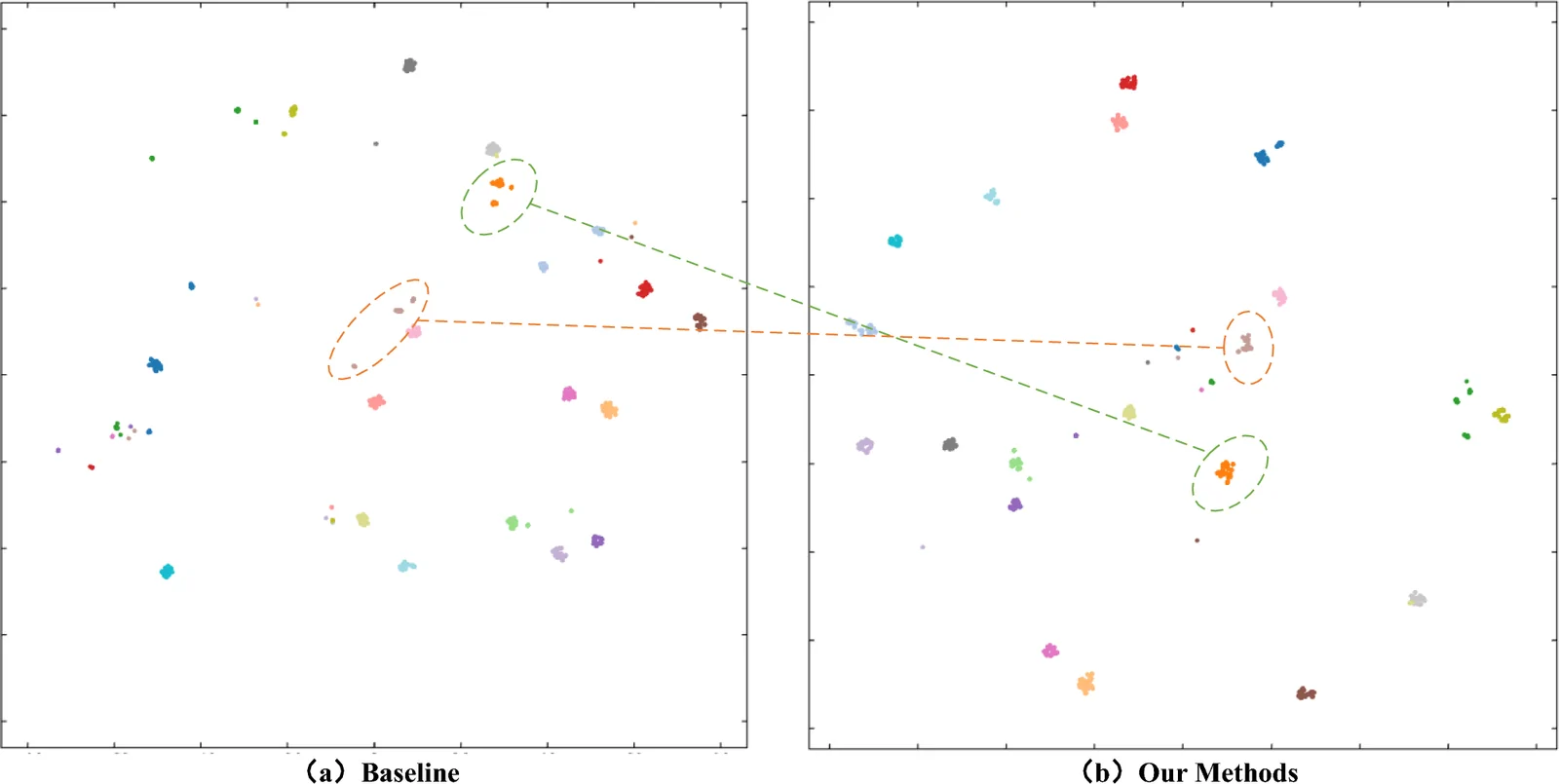
T-SNE visualization of features randomly sampled from Market1501. Different colors represent different identities. On the left, the feature clustering results of the baseline method are shown, while on the right, the feature representations obtained by our method are displayed. It is evident that the embedding space generated by our method is more discriminative and better structured.

Unsupervised person re-identification (Re-ID) can be categorized into two approaches: purely unsupervised (USL) methods and unsupervised domain adaptation (UDA) methods. UDA-based person Re-ID utilizes transfer learning or adversarial training to transfer label knowledge from a labeled source domain to an unlabeled target domain^4–6^, which enhances feature learning, especially in cross-domain scenarios^7^. However, its performance is often limited by the domain gap between the source and target. Additionally, the collection and management of labeled data can raise privacy and security concerns. In contrast, purely unsupervised Re-ID^8,9^ relies entirely on unlabeled data from the target domain. It typically uses clustering or self-supervised learning techniques to generate pseudo-labels, which are then used to train feature extractors^10^. This approach, which does not require labeled source data, enables fast deployment and offers greater generalizability and flexibility, making it increasingly appealing for practical applications. Nonetheless, generating high-quality pseudo-labels remains a critical bottleneck in improving model performance. Although traditional clustering methods can assign pseudo-labels to unlabeled data^11,12^, the inherent diversity of data and the instability of clustering algorithms often result in noisy labels. As illustrated in Fig. 1, such noise can cause intra-class dispersion and inter-class confusion, severely compromising the discriminability of the feature embedding space and limiting the model’s performance.

In order to resolve the training instability caused by unreliable pseudo-labels^13^, several recent studies have attempted to improve the reliability and robustness of pseudo-labels from the perspective of confidence modeling. Specifically, CGC^14^ estimates the Silhouette Coefficient to quantify the clustering confidence of each sample and utilizes high-confidence samples to guide centroid construction, thereby reducing the impact of noisy data. DHCCN^15^ estimates the feature distribution of samples using Gaussian Mixture Modeling and utilizes distribution-guided methods to correct the features of low-confidence samples, enhancing consistency through cross-granularity contrastive learning, thereby structurally improving the compatibility with low-confidence samples. Additionally, ACFL^16^ innovatively proposes to convert high-confidence samples into hard samples through adversarial training, creating more challenging training tasks and enhancing the model’s generalization ability in the boundary discrimination scenarios. In addition to confidence modeling, some recent studies have also focused on feature-level optimization, exploring the information interaction between global representations and local samples. CCL^17^ constructs a cluster-level memory dictionary, replacing individual samples with cluster centers for contrastive learning, effectively alleviating the issue of inconsistent instance updates and introducing a momentum update strategy to strengthen the consistency of cluster features. DCCC^18^ introduces a dynamic clustering parameter scheduler to adapt to the changes in feature distributions during training, and it designs a weight update strategy based on the distances between samples to optimize the representation of clustering centroids. These methods effectively enhance the quality of pseudo-labels and the stability of feature learning through different approaches.

Despite the significant progress made by existing methods in feature learning and pseudo-label modeling^19^, several key issues remain. Firstly, current unsupervised person re-identification methods generally lack a collaborative optimization mechanism between global and local representations^20,21^. Most approaches adopt a unidirectional optimization strategy based on cluster centers, ignoring the reverse feedback of instance features on global representations. As a result, these methods struggle to jointly model global class discriminability and local fine-grained similarity, limiting the expressive power of the embedding space. Secondly, low-confidence samples, especially unclustered outliers, are often simply discarded to maintain the purity of pseudo-labels. However, outliers include not only those extremely sparse samples that lie far from all cluster centers, but also samples that are simultaneously close to multiple cluster centers and located on the fuzzy boundaries of the feature space. These samples contain important structural information and critical discriminative patterns. Current mainstream methods still lack systematic mechanisms to dynamically assess the value of low-confidence samples and guide their effective participation in training, which severely limits the completeness of pseudo-label learning and the robustness of feature representation.

To address the aforementioned issues, this paper proposes an innovative joint optimization scheme that combines the multi-level confidence outlier refinement strategy (MLCOR) and the collaborative embedding method (CEM). Specifically, the MLCOR strategy performs dynamic confidence assessment by analyzing the distance and similarity relationships between outliers and neighboring samples, and adopts a weighted voting method based on neighboring label distributions to achieve adaptive pseudo-label correction for low-confidence samples in complex feature distribution scenarios. The method can more effectively recover and utilize samples located at the fuzzy boundaries of the feature space, enrich the feature representation, reduce noise interference and clustering errors, and thus improve the quality of the pseudo-labels and the robustness of the overall learning. Specifically, the CEM dynamically balances the interaction between samples, further increasing the inter-class distance and reducing the intra-class distance. This significantly enhances the discriminability of the embedding space and improves the adaptability of the feature space to complex scenarios.

The main contributions of this paper are as follows:

1. We design a confidence-guided outlier refinement strategy that evaluates the confidence of outliers and adopts a hierarchical processing approach. This effectively enables the recovery and utilization of outliers, significantly improving the accuracy of pseudo-labels and the robustness of the model.
2. We propose a collaborative embedding optimization method that integrates instance-level features with cluster centroids to facilitate feature interaction. By collaboratively optimizing local and global embeddings, this method achieves fine-grained similarity learning and global discriminative representation. This flexible supervision mechanism significantly enhances the discriminability and robustness of feature representations.
3. Experimental results show that the proposed method effectively recovers boundary outliers, significantly improving clustering accuracy and the discriminability of the embedding space. The method demonstrates outstanding performance on multiple datasets, surpassing most existing approaches.

## Related works

### Unsupervised person Re-ID

Unsupervised person re-identification methods are generally categorized into two types: unsupervised domain adaptation (UDA) and purely unsupervised learning (USL), with the core difference being whether source-domain annotations are utilized. UDA^22^ methods leverage labeled source data to facilitate target-domain adaptation, whereas USL^23^ approaches rely solely on unlabeled target-domain data.

Early work such as SPCL^24^ introduced adaptive contrastive learning together with a hybrid memory mechanism, gradually incorporating pseudo-labels while storing both source- and target-domain features to enhance joint representation learning. P2LR^25^ proposes a pseudo-label optimization strategy driven by probabilistic uncertainty. It quantifies the confidence of each pseudo-label and progressively incorporates only the most reliable samples through an alternating convex-search procedure, thereby balancing target-domain exploration against the detrimental effects of label noise. Some studies further address camera-specific biases^26–28^. For example, CASTOR^29^ introduces camera-shift adaptation techniques and an outlier recycling mechanism to mitigate clustering errors caused by viewpoint variations across cameras. In addition, some methods further improve performance from the perspective of pseudo-label optimization. MMT^6^ adopts a mutual mean-teaching framework, in which two networks supervise each other to progressively refine pseudo-labels and improve the robustness of feature learning. Finally, UMDA^30^ exploits multiple source domains by fusing their features and refining pseudo-labels in unison, achieving superior adaptation performance in the target domain.

USL methods often rely on clustering to generate pseudo-labels, and further incorporate various strategies to enhance the quality of these pseudo-labels and the representational capacity of learned features. MDPR^31^ introduces a mutual distillation framework that leverages multi-view and contrastive learning to distill knowledge across network branches. By adaptively refining and sharing pseudo-labels, it enhances feature consistency. DiDAL^32^ proposes a differential-aware learning approach combined with graph-based modeling, effectively improving the discriminability of identity-related features. GEPP^33^ employs generative adversarial networks to produce positive samples with viewpoint variations and optimizes positive-negative pair selection in contrastive learning through an improved sampling strategy. CHCR^34^ designs cross-modal hierarchical clustering and outlier refinement methods to optimize label assignment and feature learning in unsupervised VI-ReID. These USL approaches significantly boost the accuracy and robustness of unsupervised person re-identification by leveraging techniques such as contrastive learning, generative modeling, differential-aware learning, and pseudo-label refinement.

### Clustering algorithms

The basic idea of clustering algorithms is to partition the original data into different clusters based on distance or other criteria, without any label information. K-Means^35^ and DBSCAN^36^ are two classic clustering methods that group samples into different clusters based on distance. K-Means is suitable for data with regular cluster shapes and significant differences between clusters, while DBSCAN can effectively handle noise and irregular clusters. Both methods are simple and efficient.

PCL^37^ proposes an innovative framework. It uses K-means clustering to identify the prototype distribution of data. By modeling the relationships between prototypes and samples, PCL effectively learns sample representations and reformulates the network training process within an Expectation-Maximization framework. In practice, the DBSCAN clustering algorithm has been more widely used due to its ability to handle irregular clusters without specifying the number of clusters. Lou and colleagues^38^ employ DBSCAN to generate pseudo-labels and introduce self-supervised learning to improve label consistency, leading to better performance in unsupervised person re-identification. In DKD-MPL^39^, DBSCAN assigns labels to data from different camera views, while a dual knowledge distillation strategy guides the model to learn more reliable multi-view pseudo-labels, reducing supervision errors. However, DBSCAN has limitations, particularly in coping with continuously evolving feature distributions during training. To address this, DCCC^18^ introduces a dynamic clustering approach in which the eps parameter is adaptively adjusted throughout training to better accommodate feature distribution shifts at different stages.

### Memory dictionary

In unsupervised person re-identification, memory dictionaries play a crucial role in storing and updating feature representations, helping the model better learn from unlabeled data and effectively reducing the interference of label noise during the training process. Many studies have significantly improved the performance of unsupervised person re-identification through innovative memory dictionary designs. Typically, the average feature vector calculated from samples within a cluster is used as the centroid, while RTM^40^ updates the memory store with randomly sampled instance features, reducing feature drift and preventing the learning of fake distributions with mean-based distributions. This approach provides a novel idea for person re-identification. Some methods^41,42^ construct both cluster-level and instance-level dictionaries, combining these two types of dictionaries to enhance the model’s adaptability in handling complex data distributions. CGC^14^ constructs more stable cluster centroids by leveraging high-confidence samples. These centroids are stored in a memory dictionary, and pseudo-labels are refined through confidence-based evaluation, thereby enhancing centroid accuracy and reducing clustering errors. O2CAP^43^ utilizes a proxy memory bank to store representative information for each cluster. Pseudo-labels are generated through offline clustering combined with online feature updates, and further refined via a proxy association strategy, enhancing the stability of pseudo-labels and the robustness of the model.

## Methodology

**Fig. 2:**
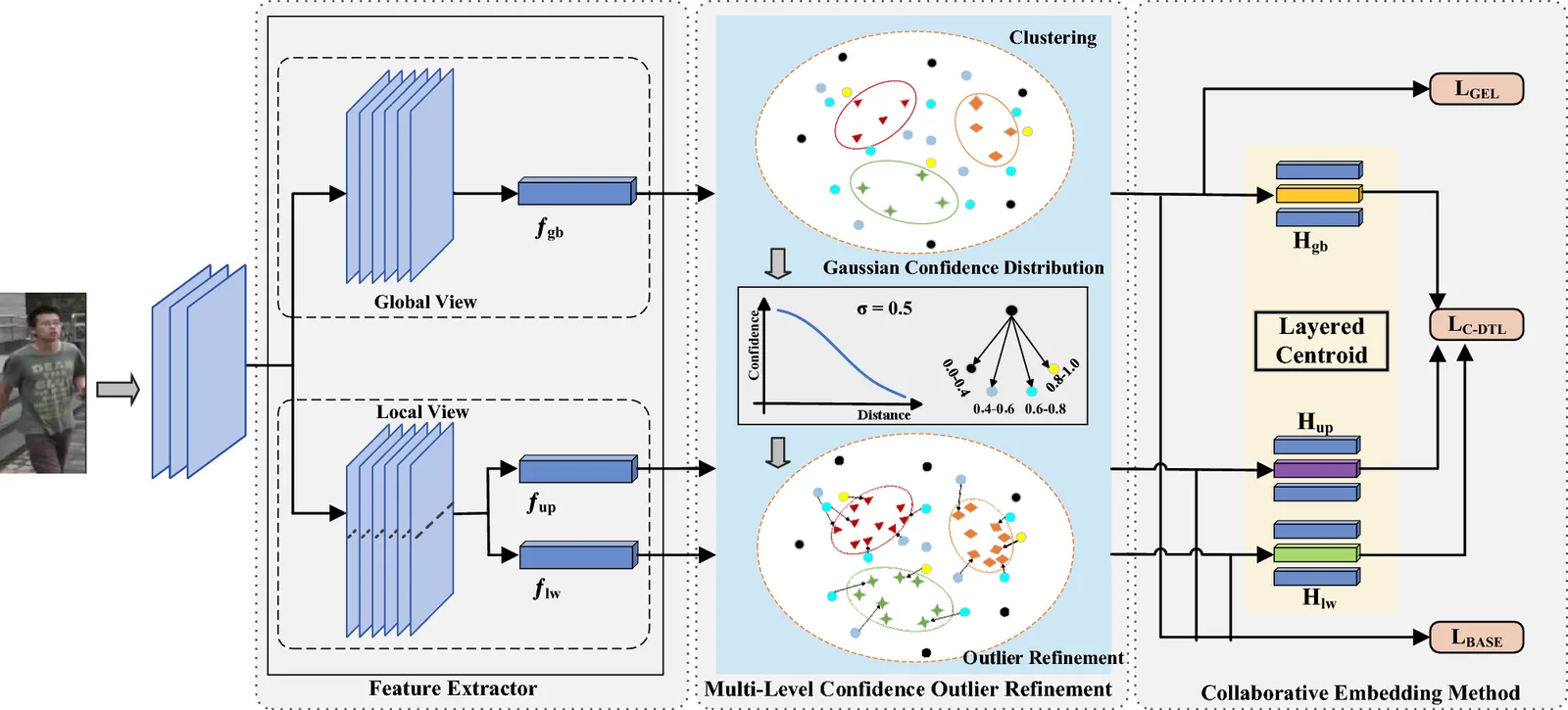
The framework of our method. (**a**) Feature Extractor: It extracts global features $f_{gb}$ and obtains multi-view features through segmentation, specifically upper-level $f_{up}$ and lower-level features $f_{lw}$. (**b**) Multi-level Confidence Outlier Refinement: The outliers are categorized into four levels and processed in a differentiated manner. Combined with iterative clustering, this approach enables the effective recovery and label correction of target outliers, which are then reincorporated into the network training pipeline for utilization. (**c**) Collaborative Embedding Method: This method combines instance-level features with cluster center information for feature interaction, and utilizes shared pseudo-labels to guide the initialization of both local and global features, constructing a more discriminative embedding space.

In this section, we first define the fundamental concepts of clustering-based fully unsupervised person re-identification methods. We then present two key components of our approach: the Multi-level Confidence Outlier Refinement strategy (MLCOR) and the Collaborative Embedding Method (CEM). As illustrated in Fig. 2, MLCOR performs multi-level confidence evaluation on outliers and selectively pulls them back through iterative clustering and feature extraction, assigning them to appropriate categories. This process guides the unsupervised re-identification by reducing clustering errors and improving the accuracy of pseudo-labels. We further introduce the CEM, which is composed of two loss functions, $L_{GEL}$ and $L_{C-DTL}$. By integrating instance-level and cluster-level information, this method increases inter-class distances while reducing intra-class distances, optimizing feature representations at both global and local levels to achieve a more discriminative embedding space. The complete algorithmic workflow is detailed in Algorithm 1.

**Algorithm 1:**
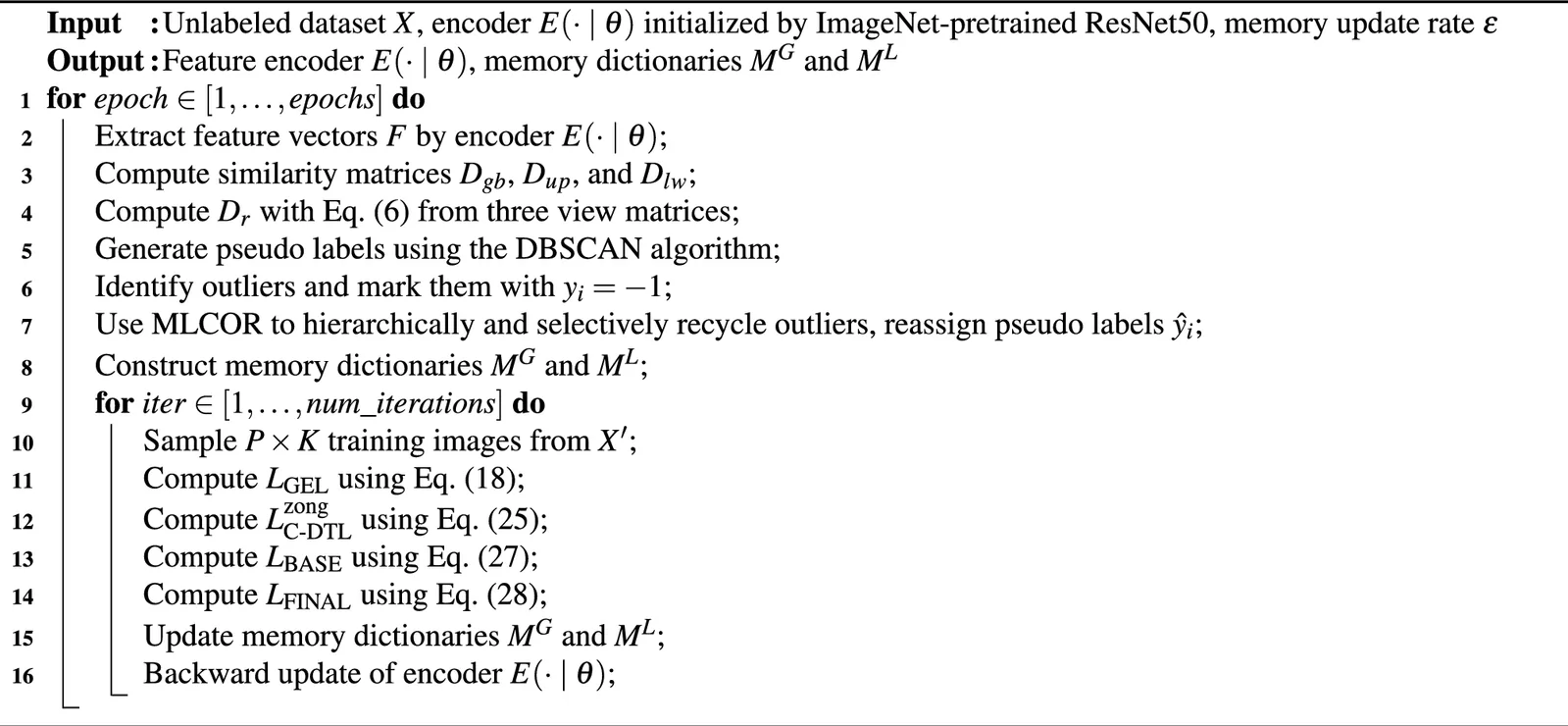
The procedure of our method.

### Premise setting

In the USL Re-ID method, we represent the unprocessed raw dataset as $X=\left\{ x_1, x_2, x_3, \ldots , x_N\right\}$, where each $x_i$ is an unlabeled pedestrian image, and the entire dataset consists of *N* pedestrian images. After being processed by the encoder $E\left( \cdot \bigg | \theta \right)$, the corresponding feature mappings $F=\left\{ f_1, f_2, f_3, \ldots , f_N\right\}$ are extracted from the dataset. where $f_i$ is the feature vector obtained from the sample $x_i$ after being processed by the encoder. We represent the global feature and the two local features as $f_{gb}$, $f_{up}$, and $f_{lw}$, respectively. Specifically, $f_{up}$ and $f_{lw}$ are obtained through spatial horizontal partitioning of the feature maps, which requires no additional computational overhead. While the global feature excels at capturing prominent holistic information, it may overlook subtle local details. The local features derived from this partitioning strategy effectively provide complementary spatial structural information, allowing both to form a more comprehensive and discriminative feature representation. Next, we calculate their Jaccard distance matrices separately: $D_{gb}$, $D_{up}$ and $D_{lw}$, and then combine them to obtain the final similarity matrix $D_r$. Pseudo-labels are assigned to all clustered samples using $D_r$ and the DBSCAN clustering algorithm, and outliers are filtered out, laying the foundation for the subsequent multi-level outlier refinement strategy. We use $\hat{y}_i$ to represent the pseudo-labels obtained for the samples after clustering. Thus, we obtain the sample pairs $U'=\left\{ (x_1,y_1), (x_2,y_2), (x_3,y_3) \ldots , (x_N,y_N)\right\}$, *C* represents the number of centroids in the clustering, the number of categories. Meanwhile, the clustering algorithm uses labels $y_i=-1$ for outliers to distinguish between cluster points and outliers, thereby achieving a more accurate partition of the internal structure of the data. Since samples have different distribution characteristics at different levels, we optimize the sample representations from both global and local perspectives to more comprehensively capture their diverse features. Generate a global instance storage dictionary $M^G$ and a local cluster storage dictionary $M^L$, $M^G$ directly store the feature vector of each training sample in sequence,while $M^L$ stores the centroids of the clusters (each centroid represents a group of similar samples). *C* denotes the number of clusters, and $m_i$ represents the centroid vector of the i-th cluster. The methods for finding centroids generally include mean-based, weighted mean-based, and random centroids. In this paper, the mean-based approach is used to calculate the centroids, which involves computing the average feature vector of all samples within the corresponding cluster. Use Eq. (1) to initialize $M^L$, with the centroids controlling the local range.

1 $$\begin{aligned} M_k^L = \frac{1}{N_k} \sum _{i \in B_k} f_i^* \end{aligned}$$

where $M_k^L$ is the centroid of the *k*-th cluster, $B_k$ is the sample set of the *k*-th cluster. * represents different levels of features, which can be $f_{gb}$, $f_{up}$, or $f_{lw}$, and $N_k$ is the number of samples in cluster *k*. On the other hand, we initialize $M_{i}^{G}$ based on Eq. (2):

2 $$\begin{aligned} M_{i}^{G} = f_i^{*} \end{aligned}$$

where the feature vector corresponding to the *i*-th training sample is directly stored in the *i*-th row of the dictionary in sequence, forming a matrix structure where each row represents the feature vector of a training sample, facilitating subsequent similarity measurement.

Unsupervised person re-identification is typically implemented within the framework of clustering-based contrastive learning^44,45^. The typical procedure of clustering-based contrastive learning in unsupervised person re-identification is generally divided into four steps: (1) Preprocess the data and extract features. (2) Generate pseudo-labels. (3) Optimize the model using a loss function. (4) Perform iterative training and evaluation. The standard cluster-level contrastive loss computes the similarity between the feature $f_i$ of the input sample and the centroid $M_{\hat{y}_i}^L$ corresponding to its assigned pseudo-label. The objective is to maximize the similarity between the sample and its corresponding cluster centroid while separating it from other cluster centroids. The sample-wise clustering loss can be expressed as follows:

3 $$\begin{aligned} L_{\textrm{cluster}}^{(i)} = -\log \frac{ \exp \left( \langle f_i, M_{\hat{y}_i}^L \rangle / \tau \right) }{ \sum _{j=1}^{C} \exp \left( \langle f_i, M_j^L \rangle / \tau \right) } \end{aligned}$$

where $f_i$ represents the feature vector of the *i*-th input sample, $\hat{y}_i$ denotes the pseudo-label assigned to this sample, $M_{\hat{y}_i}^L$ is the centroid of the cluster corresponding to $\hat{y}_i$, $M_j^L$ is the centroid of the *j*-th cluster, *C* is the total number of clusters, and *τ* is the temperature coefficient that controls the similarity distribution. While optimizing the model, it is necessary to update the local dictionary and global dictionary in real time using the input samples. The specific updating processes of $M_i^G$ and $M_k^L$ are illustrated by Eqs. (4) and (5):

4 $$\begin{aligned} M_i^G = \varepsilon M_i^G + (1-\varepsilon ) f_i \end{aligned}$$

5 $$\begin{aligned} M_k^L = \varepsilon M_k^L + (1-\varepsilon ) f_i \end{aligned}$$

where *\varepsilon* represents the update rate for both the global dictionary and the local dictionary. The two update rules are similar in form, but the main difference is that the local dictionary updates the centroid associated with the input sample, whereas the global dictionary updates only the entry corresponding to the sample index, without affecting other positive samples. This ensures that each sample retains its independent discriminative representation. In the training phase, we adopt the *PK* sampling strategy, randomly selecting *P* person IDs, and randomly picking *K* person images from each selected ID for training.

### Multi-level confidence outlier refinement

**Fig. 3:**
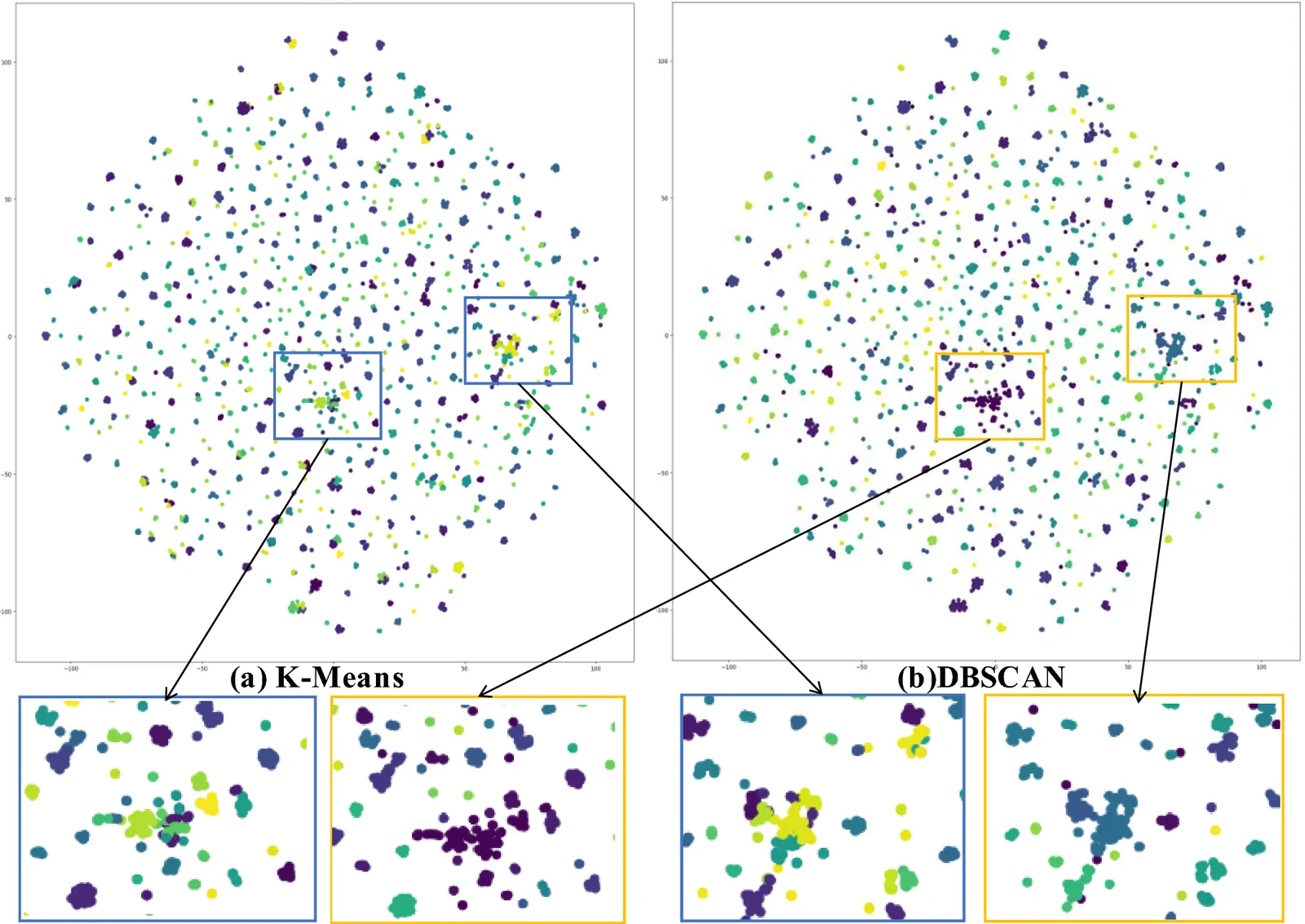
A qualitative comparison of clustering results on Market1501 using K-means and DBSCAN. The left panel corresponds to K-means (blue box), while the right panel corresponds to DBSCAN (orange box). It is evident that DBSCAN is more suitable for clusters with uneven density and irregular shapes, yielding clearer cluster boundaries.

Points with a pseudo label of *-1* after clustering are defined as outliers, denoted by $O=\{i \mid \hat{y}_i=-1\}$. We performed clustering using K-Means and DBSCAN, respectively, and compared their results, as shown in Fig. 3. Although many clustering-based unsupervised Re-ID approaches discard outliers to avoid noise propagation, outliers typically lie near cluster boundaries or transitional regions in the feature space. They include not only isolated samples far from all cluster centers, but also ambiguous samples that are close to multiple centers and thus difficult to assign. Such samples may still contain valuable structural and discriminative information. To exploit this information, we propose a multi-level confidence outlier refinement strategy that leverages neighborhood label agreement to selectively pull back outliers and correct pseudo labels, thereby improving training stability.

We first construct a fused distance matrix for neighborhood retrieval. Specifically, we compute Jaccard distance matrices based on the global feature set $F_{gb}$ and two local feature sets $F_{up}$ and $F_{lw}$, and combine them with a weighting coefficient $m_1$ to obtain the fused distance matrix ${D}_r$, as defined in Eq. (6):

6 $$\begin{aligned} D_r = (1 - 2m_1) D_{gb} + m_1 D_{up} + m_1 D_{lw} \end{aligned}$$

Then comes the step of finding reliable neighbor points. For each outlier *i*, we retrieve its top-*s* nearest neighbors ${N}_s(i)$ according to ${D}_r$. To suppress accidental one-way neighbor matches, we adopt a reciprocal neighbor constraint^46^: a neighbor *j* is considered a valid candidate only when $j\in {N}_s(i)$ and $i\in {N}_s(j)$ simultaneously hold. This reciprocal neighbor check filters unstable neighborhood relations and provides a more reliable basis for subsequent pull-back and refinement.

Next, we quantify the reliability of whether a neighbor is suitable for pull-back. For an outlier point *i* and a clustered neighbor *j* ($\hat{y}_j\ne -1$), we map the fused distance $d_{i,j}={D}_r(i,j)$ to a confidence value via a Gaussian kernel. The confidence *V* is defined in Eq. (7):

7 $$\begin{aligned} V_{i,j}=\exp \!\left( -\frac{d_{i,j}^{2}}{2\sigma ^{2}}\right) \end{aligned}$$

Here, *σ* is the standard deviation that controls the width of the Gaussian kernel; a smaller *σ* leads to faster confidence decay with increasing distance. We define the recovery reliability of outlier *i* as $V_i=\max _{j\in {N}_s(i),\,\hat{y}_j\ne -1} V_{i,j}$ over its valid clustered neighbors, and partition outliers into four confidence intervals as in Eq. (8). Samples with confidence below 0.40 are not pulled back in the current round to avoid excessive noise injection. Moreover, we set a per-round recovery budget upper bound *B* and adopt a stage-wise allocation strategy: we prioritize recovering high-confidence samples; only when the number of recovered high-confidence outliers has not reached *B*/2 do we allow medium/low-confidence samples to consume the remaining budget. Once high-confidence recoveries reach *B*/2, we stop recovering medium/low-confidence samples in the current round, thereby suppressing error propagation while ensuring sufficient reliable recovery.

8 $$\begin{aligned} V = \left\{ \begin{array}{ll} 0.8\sim 1.0 & high\_confidence \\ 0.6\sim 0.8 & medium\_confidence \\ 0.4\sim 0.6 & low\_confidence \\ 0.0\sim 0.4 & outlier \end{array} \right. \end{aligned}$$

For high/medium-confidence outliers ($V_i \ge 0.60$), neighborhood similarity is stronger and the risk of label transfer is relatively low. We thus adopt direct inheritance, and update pseudo labels according to Eq. (9):

9 $$\begin{aligned} j_i=\arg \max _{j\in {N}_s(i),\,\hat{y}_j\ne -1} V_{i,j} \end{aligned}$$

update its pseudo-label $\hat{y}_i$ to $\hat{y}_{j_i}$, where $\arg \max$ returns the neighbor index that maximizes $V_{i,j}$.

For low-confidence outliers ($0.40 \le V_i < 0.60$), samples often lie in transitional regions between clusters or exhibit comparable similarity to multiple clusters; directly inheriting a single neighbor label may introduce noise. We therefore perform weighted neighbor voting to extract more reliable cues from the local label distribution. We first define the valid neighbor set in Eq. (10) and compute the weighted score for each candidate class *k* using Eq. (11):

10 $$\begin{aligned} {N}^{\ell }(i)=\{j\in {N}_s(i)\mid \hat{y}_j\ne -1\} \end{aligned}$$

11 $$\begin{aligned} \text {score}_{k}(i)=\sum _{j\in {N}^{\ell }(i)} \exp \!\left( -\frac{d_{i,j}^{2}}{2\sigma ^{2}}\right) \cdot \mathbb {I}(\hat{y}_{j}=k) \end{aligned}$$

We then set $k_i=\arg \max _k \text {score}_{k}(i)$. To ensure sufficient local agreement, we impose the consensus constraint in Eq. (12):

12 $$\begin{aligned} \frac{\text {score}_{k_i}(i)}{\sum _{k}\text {score}_{k}(i)} \ge \rho \end{aligned}$$

where *ρ* denotes the threshold for accepting the weighted voting result. The pseudo label is updated to $k_i$ only when Eq. (12) holds; otherwise, we keep $\hat{y}_i=-1$. With the above stratified updating strategy, we prioritize recovering reliable outliers while applying conservative weighted voting and consensus checking to uncertain samples, which suppress noise accumulation and improves the stability of pseudo-label refinement. Samples with $V_i<0.40$ or those failing the recovery conditions remain outliers ($\hat{y}_i=-1$) and are revisited in subsequent rounds. As pseudo labels are progressively updated and neighborhood structures are reshaped, a subset of outliers can obtain stable labels and be merged into clusters. Our approach evaluates outlier reliability via neighborhood consistency, performs confidence-aware recovery, and refines pseudo labels, thereby enhancing the robustness of model training.

### Collaborative embedding method

In the unsupervised person re-identification task, existing methods mainly focus on pseudo-label learning, self-training, contrastive learning^47,48^ and knowledge distillation^49,50^. Although these approaches partially alleviate label scarcity, significant shortcomings remain in balancing global feature consistency and local discriminative ability. Cluster-based pseudo-label methods generate identity labels by clustering features and optimize them using loss functions. However, these methods heavily rely on the quality of the current model’s features. During the early stages of training, the clustering results are susceptible to interference from outliers and noisy samples, leading to unstable pseudo-labels that affect model convergence. Contrastive learning enhances feature discriminability by constructing positive and negative sample pairs, but in the case of unreliable pseudo-labels, it is prone to introducing incorrect supervision signals, which causes a shift in the training objective. Knowledge distillation alleviates pseudo-label errors by introducing a teacher model, but it comes with additional computational overhead and synchronization challenges. Moreover, it is fundamentally still constrained by clustering quality and cannot fundamentally eliminate the impact of pseudo-label noise.

**Fig. 4:**
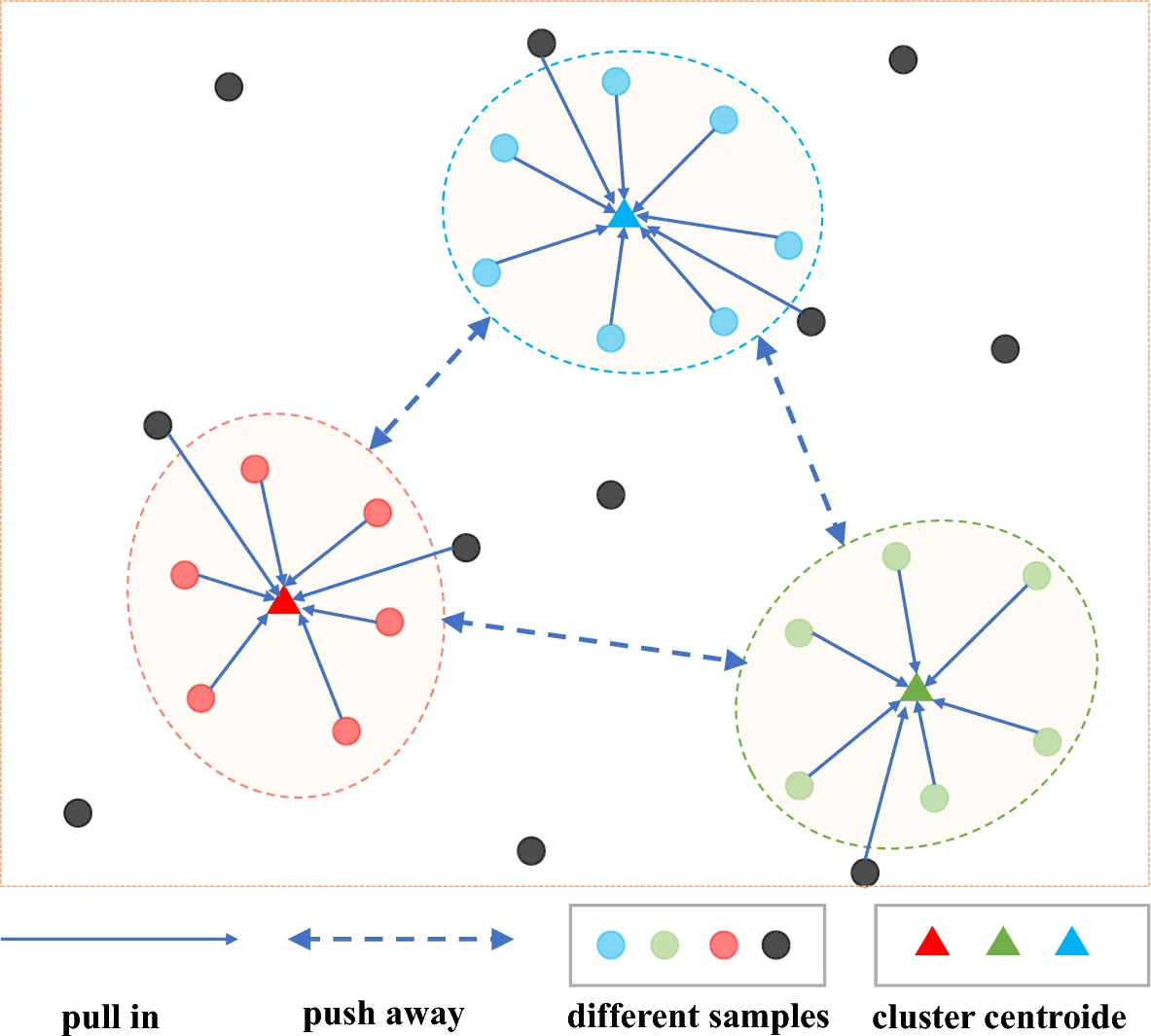
The embedding space. Different colored circles represent different identities, while different colored triangles represent distinct centroids. Black circles denote outliers. We recover some highly similar outliers for further use, while simultaneously leveraging CEM to ensure that similar samples are brought closer together, and dissimilar samples are pushed farther apart.

In this context, we propose CEM that combines Global Embedding Loss $(L_{GEL})$ and Cluster-Driven Triplet Loss $(L_{C-DTL})$. This approach not only improves the global feature modeling ability but also enhances the discriminative power of local features, optimizing the embedding space, as shown in Fig. 4. Additionally, as discussed in the previous section, it effectively reduces the interference of outliers during their recovery process, ensuring minimal disruption to the training.

#### Global embedding loss

In unsupervised person re-identification, pseudo-label noise and mini-batch sampling bias often weaken global structural constraints in the embedding space, leading to dispersed intra-class features and ambiguous inter-class boundaries. To address this issue, we introduce the Global Embedding Loss, denoted as $L_{GEL}$. By leveraging a global memory bank as a cross-iteration reference, $L_{GEL}$ enables each sample to be optimized against an approximate full data distribution context, thereby improving intra-class compactness, inter-class separability, and overall training stability.

During training, we maintain a global feature memory bank ${M}_G \in {R}^{N \times D}$, where *N* denotes the total number of training samples and *D* denotes the feature dimension. The feature stored for the *j*-th instance is denoted as ${m}_j$, with its pseudo-identity label $y_j \in \{1,2,\ldots ,C\}$. For the *i*-th input sample feature ${x}_i$ in a mini-batch, we compute its similarity responses to all instances in the memory bank, as shown in Eq. (13):

13 $$\begin{aligned} {z}_i=\frac{{sim}({x}_i,{M})}{\tau }, \qquad \tilde{{z}}_i={z}_i-\max ({z}_i) \end{aligned}$$

where *τ* is a temperature scaling factor and $\max (\cdot )$ shifts ${z}_i$ by its maximum component for numerical stability. Consequently, $\tilde{{z}}_i\in {R}^{N}$ represents the responses of ${x}_i$ to individual memory instances. To obtain a class-level global representation, we one-hot encode the pseudo-label vector of the memory bank and construct the class indicator matrix ${Y}\in \{0,1\}^{N\times C}$, as defined in Eq. (14):

14 $$\begin{aligned} {Y}=\mathrm {one\_hot}({y}, C) \end{aligned}$$

Next, we apply an exponential mapping to the stabilized logits and aggregate the instance-level evidence within each pseudo-identity class, yielding a class-wise aggregated score vector ${S}_i\in {R}^{C}$, as formulated in Eq. (15):

15 $$\begin{aligned} {S}_i=\exp (\tilde{{z}}_i)^{\top }{Y} \end{aligned}$$

The *c*-th component of ${S}_i$ (the aggregated score of sample *i* for pseudo-identity *c*) can be written explicitly as Eq. (16):

16 $$\begin{aligned} S_{i,c}=[{S}_i]_c=\sum _{j:\,y_j=c}\exp (\tilde{z}_{i,j}) \end{aligned}$$

Here, $y_j$ denotes the pseudo-label of the *j*-th memory instance, and $\tilde{z}_{i,j}$ is the stabilized response between sample *i* and memory instance *j*. This aggregation accumulates multi-instance evidence within the same pseudo-identity: if ${x}_i$ is consistently similar to multiple instances of a class, $S_{i,c}$ becomes large; otherwise it remains small, which helps reduce sensitivity to noisy individual instances.

Since some pseudo-identity classes may be absent in the memory bank, we introduce a validity mask to prevent empty classes from participating in the normalization, as formulated in Eq. (17):

17 $$\begin{aligned} \textrm{mask}_c=\mathbb {I}(\textrm{count}_c>0) \end{aligned}$$

where $\textrm{count}_c$ is the number of memory instances assigned to class *c*. Finally, we normalize $\{S_{i,c}\}$ over valid classes only and maximize the normalized aggregated response for the target pseudo-label $y_i$ via a negative log-likelihood objective, $L_{GEL}$ can be expressed as Eq. (18):

18 $$\begin{aligned} {L}_{{GEL}} = -\frac{1}{B}\sum _{i=1}^{B} \log \frac{S_{i,y_i}\cdot \textrm{mask}_{y_i}}{\sum _{c=1}^{C} S_{i,c}\cdot \textrm{mask}_{c}} \end{aligned}$$

Here, $y_i$ is the target pseudo-identity label of sample *i*. The loss function first generates responses for each instance based on the memory bank, and then aggregates these responses by pseudo-identity categories, followed by normalization over the valid category set. In this way, the model simultaneously optimizes feature aggregation for the same identity and feature separation between different identities from a global perspective, significantly improving the discriminability of the embedding space and the stability of the training process.

#### Cluster-driven triplet loss

Although the global embedding loss helps optimize the feature distribution across classes, the fine-grained differences and local structures in person re-identification tasks are still difficult to fully capture through global optimization alone, resulting in limited perception and discriminative capability in boundary regions. To address this, we introduce the Cluster-Driven Triplet Loss $L_{C-DTL}$ to enhance local structure modeling and improve model robustness.

Unlike traditional Triplet Loss, which simultaneously imposes constraints on both positive and negative samples, our approach focuses exclusively on pushing apart hardest negative samples, intentionally neglecting the forced compression of hardest positive sample distances. This is done to mitigate the structural interference caused by potential pseudo-label errors. The key motivation is that, under clustering-based pseudo labels, the hardest positive may be a false-positive sample; forcibly pulling such pairs closer can distort the local structure and exacerbate error propagation. In contrast, the hardest negative is more likely to come from a different cluster, featuring clearer semantics and a lower error rate. Therefore, prioritizing negative-pair separation helps stabilize training and strengthen inter-class boundaries.

Concretely, given an anchor feature $f_i$, we mine the hardest positive $f_{p(i)}$ and the hardest negative $f_{n(i)}$ in the current embedding space. To obtain a more stable supervisory signal, we introduce a reference representation $f^{ref}$ to construct a soft-label distribution. The reference representation is generated by the same network at the current iteration and is stop-gradient in the loss, so that it provides soft-supervision weights only without participating in back-propagation, thereby improving training stability. The anchor–negative soft label in the reference space is defined as Eq. (19):

19 $$\begin{aligned} P_{an}^{(i)} = \frac{ \exp \!\left( -\left\| f_i^{ref}-f_{n(i)}^{ref}\right\| _2\right) }{ \exp \!\left( -\left\| f_i^{ref}-f_{p(i)}^{ref}\right\| _2\right) +\exp \!\left( -\left\| f_i^{ref}-f_{n(i)}^{ref}\right\| _2\right) } \end{aligned}$$

The corresponding log-probability (log-softmax) in the online feature space is defined as Eq. (20):

20 $$\begin{aligned} Q_{an}^{(i)}=\log \left( \frac{\exp \!\left( -\left\| f_i-f_{n(i)}\right\| _2\right) }{\exp \!\left( -\left\| f_i-f_{p(i)}\right\| _2\right) +\exp \!\left( -\left\| f_i-f_{n(i)}\right\| _2\right) } \right) \end{aligned}$$

To further suppress the interference of outlier samples during training, we design a weighting mechanism based on cluster centroids. This mechanism dynamically assigns sample weights by evaluating the distance between each sample and its nearest cluster centroid. The feature representations used for centroid construction are summarized in Eq. (21):

21 $$\begin{aligned} \left\{ \begin{aligned} {F_{gb}}=\left\{ {f_{gb}^1},{f_{gb}^2},{f_{gb}^3},...,{f_{gb}^N}\right\} \\ {F_{up}}=\left\{ {f_{up}^1},{f_{up}^2},{f_{up}^3},...,{f_{up}^N}\right\} \\ {F_{lw}}=\left\{ {f_{lw}^1},{f_{lw}^2},{f_{lw}^3},...,{f_{lw}^N}\right\}&\end{aligned} \right. \end{aligned}$$

Then, based on the pseudo-labels, we perform independent clustering operations on each branch, initializing the local dictionary using Eq. (1). The final result is a matrix representing the clustering centroids at three levels, as shown in Eq. (22):

22 $$\begin{aligned} \left\{ \begin{aligned} {H_{gb}}=\left\{ {h_{gb}^1},{h_{gb}^2},{h_{gb}^3},...,{h_{gb}^C}\right\} \\ {H_{up}}=\left\{ {h_{up}^1},{h_{up}^2},{h_{up}^3},...,{h_{up}^C}\right\} \\ {H_{lw}}=\left\{ {h_{lw}^1},{h_{lw}^2},{h_{lw}^3},...,{h_{lw}^C}\right\}&\end{aligned} \right. \end{aligned}$$

Here, *C* denotes the number of clusters. The three branches share the same set of pseudo labels; thus, the number of cluster categories remains consistent across them. Based on the above cluster centroids, sample weights $p_i$ are further introduced to measure the structural reliability of each sample. These weights are later employed in the loss function to guide the model to focus on structurally reliable sample regions. Each weight is determined by the minimum distance between a sample and all cluster centers. Specifically, the sample weight is inversely correlated with the distance to its nearest cluster center. It should be noted that the nearest cluster center is used here, rather than the assigned centroid of the sample. The formulation is given in Eq. (23):

23 $$\begin{aligned} p_i = \exp \left( - \frac{1}{2} \min \limits _{c \in C} \parallel f_i - h_c \parallel _2 \right) \end{aligned}$$

where $f_i$ represents the feature of the *i*-th sample, $h_c$ denotes the centroid of the *c*-th cluster, and *C* is the set of all cluster indices. This weighting mechanism is intended to measure the structural reliability of a sample in the embedding space, rather than to enforce consistency with its assigned pseudo label. It helps reduce the influence of outliers and improves the model’s ability to capture local structures.

From this, $L_{C-DTL}$ can be defined as Eq. (24). The sum of multi-level losses, $L_{C-DTL}^{zong}$, is defined in Eq. (25):

24 $$\begin{aligned} {L}_{C-DTL}^{*} = \frac{1}{N} \sum _{i=1}^{N} p_i \cdot \left( -P_{an}^{(i)} \cdot \log Q_{an}^{(i)} \right) \end{aligned}$$

25 $$\begin{aligned} L^{zong}_{C-DTL} = L^{gb}_{C-DTL} + \beta \left( L^{up}_{C-DTL} + L^{lw}_{C-DTL} \right) \end{aligned}$$

Here, * indicates the different levels of loss, which can be taken as $f_{gb}$, $f_{up}$ and $f_{lw}$. The coefficient *β* is used to regulate the contribution of each component to the total loss. By integrating multi-scale information and introducing clustering guidance, $L^{zong}_{C-DTL}$ enhances discriminative capability while effectively reshaping inter-class boundaries in the embedding space. In addition, this design complements the proposed MLCOR strategy: while MLCOR is responsible for mining and correcting potential outlier positive samples within pseudo labels, the proposed loss function focuses on supervising reliable negative pairs and incorporates a clustering-based weighting mechanism to mitigate the interference of extremely hard negatives during optimization. The collaboration between the two contributes to constructing a more stable and structured feature space under complex data distributions.

#### Baseline loss

In addition to the aforementioned loss terms, we further adopt the baseline cross-entropy loss for optimization. Cross-entropy loss is widely used in classification tasks, especially for multi-class problems, as it measures the discrepancy between the predicted probability distribution and the ground-truth label distribution. A smaller cross-entropy loss indicates that the predicted distribution is closer to the true distribution. The cross-entropy loss at each level can be formulated as:

26 $$\begin{aligned} L_{CE}^{*} = -\frac{1}{N} \sum _{i=1}^{N} \log \left( \frac{\exp (f_i^T W_{y_i})}{\sum _{c=1}^{C} \exp (f_i^T W_c)} \right) , \end{aligned}$$

where *N* denotes the number of samples, *C* denotes the number of classes, $f_i$ is the feature vector of sample *i*, $W_c$ is the class prototype (or classifier weight) of class *c*, and $y_i$ is the ground-truth label of sample *i*. Here, $* \in \{gb, up, lw\}$ denotes different levels. The overall baseline loss is defined as Eq. (27), where $m_2$ is the balancing coefficient used to regulate the contributions of loss terms from different levels.

27 $$\begin{aligned} L_{BASE} = (1 - m_2) L_{CE}^{gb} + m_2 \left( L_{CE}^{up} + L_{CE}^{lw} \right) , \end{aligned}$$

Finally, all loss terms are integrated to jointly optimize the model, as expressed in Eq. (28), where $w_1$ and $w_2$ are balancing coefficients used to control the contribution of each loss term:

28 $$\begin{aligned} L_{FINAL} = L_{BASE} + w_1 L_{C\text {-}DTL}^{zong} + w_2 L_{GEL}, \end{aligned}$$

## Experiments

### Dataset and evaluation protocol

We conducted extensive experiments on three datasets: the person Re-ID datasets Market1501^51^, DukeMTMC-reID^1^ and MSMT17^52^. Detailed information about the datasets is shown in Table 1.

**Table 1:** We provide detailed statistics on three person Re-ID datasets.

Datasets	Scene	Training		Gallery		Query		Cams
		Pids	Imgs	Pids	Imgs	Pids	Imgs	
Market1501	Outdoor	751	12936	750	15913	750	3368	6
DukeMTMC-reID	Outdoor	702	16522	1110	17661	702	2228	8
MSMT17	Outdoor, Indoor	1057	32621	3060	82161	3060	11659	15

**Market1501:** One of the most widely used person re-identification datasets, Market1501 was released by researchers from Tsinghua University in 2015. The dataset consists of 32,688 images of 1,501 identities captured by six cameras. Of these, 12,936 images from 751 identities are used for training, while the remaining 19,732 images from 750 identities form the test set. The query set consists of 3,368 images from 750 identities. All images in Market1501 were captured in the same shopping mall, making it a simple and widely used dataset in academic research.

**DukeMTMC-reID**^53^: Provided by Duke University, this dataset is captured by eight cameras and contains 36,411 images of 1,404 identities. The training set includes 16,522 images from 702 identities, while the test set consists of 19,889 images from 702 identities. This dataset covers diverse scenes, which increases the difficulty of matching.

**MSMT17:** Released by Microsoft Research Asia, this dataset contains 126,441 images of 4,101 individuals. The images in this dataset cover various weather conditions, times of day, and viewpoints, with complex backgrounds. It is highly challenging and mainly used to address complex and diverse person matching problems.

**Protocols:** Person re-identification is an image retrieval task. We evaluate model robustness using Mean Average Precision (mAP) and Cumulative Matching Characteristic (CMC) to assess our method’s performance on three benchmark datasets. During testing, no post-processing operations, such as re-ranking, were applied.

### Implementation details

We use ResNet50, pre-trained on ImageNet, as the backbone for all experiments, with training and testing conducted on two NVIDIA A40 GPUs. Specifically, we remove all modules after the fourth convolutional layer in the backbone network and add a Generalized Average Pooling (GEM) layer, followed by a batch normalization layer and an L2 normalization layer. The output is a 2048-dimensional vector representation. We use DBSCAN for clustering and apply data augmentation to the images via random horizontal flipping, cropping, and erasing. The images from Market1501, DukeMTMC-reID, and MSMT17 are resized to *256× 128*, with their respective eps values set to 0.6, 0.7, and 0.7. During training, we use the Adam optimizer with a weight decay of 5e-4 for model updates. The initial learning rate is set to 3.5e-4 and decayed every 20 epochs. The whole training process lasts for 50 epochs with 200 iterations per epoch. In the proposed MLCOR strategy, the fusion coefficient $m_1$ in Eq. (6) is set to 0.15, and the consensus threshold *ρ* in Eq. (12) is set to 0.6. The number of retrieved nearest neighbors *s* is set to 5 on Market1501 and to 7 on DukeMTMC-reID and MSMT17. The standard deviation *σ* is set to 0.5, and the per-round outlier recovery budget upper bound *B* is set to 40. In addition, other hyperparameters such as *σ*, *s*, and *B* are related to the MLCOR mechanism. These parameters mainly control the smoothness of confidence estimation, the number of retrieved neighbors, and the recovery budget, respectively. Among them, *σ* and *B* are fixed to empirically effective values, while *s* is adjusted within a small range according to the data scale and neighborhood density of each dataset. In practice, we observe that the model performance remains stable within reasonable ranges of these parameters. The global and local memory update rates, *\varepsilon*, are set to 0.1 in Eq. (4) and Eq. (5), with the balancing factor *β* set to 0.2 in Eq. (25). The loss balancing coefficient $m_2$ in Eq. (27) is set to 0.2. The balancing factor $w_2$ is set to 0.6 in Eq. (28), while $w_1$ is set to 1.0, 0.8, and 0.4 for the Market1501, DukeMTMC-reID, and MSMT17 datasets, respectively, according to the sensitivity analysis shown in Fig. 6d–f.

**Table 2:** We compare the proposed method with various algorithms on Market1501, MSMT17, and DukeMTMC-reID datasets. Bold and italic values represent the best and second-best results, respectively. “–” indicates that the results are not provided.

Methods	Reference	Market1501				MSMT17				DukeMTMC-reID			
		R1	R5	R10	mAP	R1	R5	R10	mAP	R1	R5	R10	mAP
SpCL^24^	NeurIPS20	88.1	95.1	97.0	73.1	42.3	55.6	61.2	19.1	81.2	90.3	92.2	65.3
IICS^27^	CVPR21	89.5	95.2	97.0	72.9	56.4	68.8	73.4	26.9	80.0	89.0	91.6	64.4
Cluster^17^	ACCV22	92.9	97.2	98.0	83.0	62.0	71.8	76.7	33.0	–	–	–	–
IIDS^54^	TPAMI22	91.2	96.2	97.7	78.0	64.4	76.2	80.5	35.1	82.1	90.8	93.7	68.7
O2CAP^43^	TIP22	92.5	–	–	82.7	72.0	–	–	*42.4*	83.9	–	–	71.2
NNNI^10^	TCSVT23	93.5	97.4	98.3	84.3	64.0	74.8	79.0	35.3	86.2	92.4	94.6	74.1
GroupSampling^19^	TCSVT23	92.3	96.6	97.8	79.2	56.2	67.3	71.5	24.6	82.7	91.1	93.5	69.1
RTMem^40^	TIP23	92.8	97.4	98.3	83.0	57.1	70.0	74.9	32.8	–	–	–	–
DHCCN^15^	TCSVT24	94.1	–	–	85.6	65.9	–	–	36.4	83.8	–	–	73.4
PISL^20^	TIP24	92.4	96.8	97.6	81.1	62.3	74.6	78.6	34.9	–	–	–	–
STDA^13^	TITS24	93.1	97.3	98.4	82.7	62.6	73.4	77.5	31.8	–	–	–	–
HCACE^26^	TMM24	93.7	97.5	98.1	83.4	–	–	–	–	84.1	91.9	94.2	71.5
MPRD^31^	TNNLS24	*94.6*	97.8	*98.7*	85.8	67.2	78.3	82.3	39.1	*86.3*	92.4	*94.8*	*75.5*
RPE^3^	TMM24	92.6	97.1	97.9	82.4	–	–	–	–	77.8	89.3	91.7	71.5
CGC^14^	TIFS24	94.2	97.6	98.5	85.3	63.4	74.6	79.3	34.6	–	–	–	–
DCCC^18^	ICASSP24	94.1	**98.0**	**98.9**	*86.6*	62.3	73.4	77.9	31.6	–	–	–	–
TIG-CL^47^	IOTJ24	**94.7**	*97.9*	98.5	*86.6*	**69.6**	*79.0*	*82.7*	39.2	86.2	*93.0*	94.7	75.2
Multi Queue^48^	ICASSP25	92.8	96.9	98.0	82.8	64.7	75.4	79.3	34.7	84.6	91.9	94.1	72.0
DSFNet^55^	IF26	94.6	97.3	–	86.7	67.1	74.6	–	35.7	–	–	–	–
Ours		94.4	*97.9*	98.6	**86.8**	*68.5*	**80.0**	**83.7**	**43.6**	**86.4**	**93.4**	**95.4**	**75.7**

### Comparison with the state-of-the-art methods

We conducted extensive comparisons between our method and various unsupervised person re-identification methods on the Market1501, DukeMTMC-reID and MSMT17 datasets, with the results shown in Table 2. The results demonstrate that our method is highly competitive across all datasets.

The results show that our method achieves the best mAP on Market1501. However, some methods still perform strongly on certain metrics. In the 2024 study, TIG-CL^47^ achieved 94.7% Rank-1 and 97.9% Rank-5 accuracy. It proposed a teacher-guided contrastive learning framework that combines individual awareness and group awareness, enhancing feature representation through group-level probability distillation and individual-level relationship distillation. Additionally, MPRD^31^ achieved 94.6%, 97.8% and 98.7% Rank-1, Rank-5 and Rank-10 scores, respectively. It addresses the performance degradation caused by uncertain clustering numbers by introducing a Meta pairwise relationship distillation method, which determines the relationships between sample pairs based on their neighborhood graph structure, achieving higher retrieval performance. We achieved the best performance across all metrics on DukeMTMC-reID. Compared with Multi Queue^48^, our Rank-1 improved by 1.8%, and mAP increased by 3.7%. Compared to the second-best MPRD, our method shows improvements in all metrics, and, unlike IIDS^54^ and CAP^56^, we do not require camera information. On MSMT17, we achieved high performance as well. Compared to the second-best mAP, O2CAP, our method improved by 1.2%. Various methods aim to optimize the model’s robustness in complex scenarios. GroupSampling^19^ introduces an innovative group sampling technique to enhance the statistical stability of feature representations, while O2CAP^43^ captures the complex relationships between samples and pseudo-labels by selecting appropriate positive and negative sample sets. Most methods overlook outliers, while we recover key boundary outliers to learn boundary determination information and construct a more discriminative embedding space using the CEM. This enables our method to demonstrate better robustness and adaptability in complex scenarios.

**Table 3:** We conduct an ablation study of our method, performing separate ablation studies on $L_{C-DTL}^{zong}$ and $L_{GEL}$ of CEM.

Method			Market1501		MSMT17		DukeMTMC	
MLCOR	$L_{C-DTL}^{zong}$	$L_{GEL}$	mAP	R1	mAP	R1	mAP	R1
–	–	–	83.9	93.3	34.2	61.0	70.5	82.9
*\checkmark*	–	–	84.3	93.9	35.3	62.6	72.3	84.5
-	*\checkmark*	–	84.6	93.5	35.8	62.6	72.5	83.9
–	–	*\checkmark*	85.4	93.8	42.4	67.8	73.7	85.1
*\checkmark*	*\checkmark*	–	85.6	94.0	36.0	62.7	73.0	85.2
–	*\checkmark*	*\checkmark*	86.4	94.0	43.3	67.8	75.0	85.5
*\checkmark*	–	*\checkmark*	85.6	93.8	43.0	68.4	73.9	85.2
*\checkmark*	*\checkmark*	*\checkmark*	86.8	94.4	43.6	68.5	75.7	86.4

### Ablation study

In order to evaluate the effectiveness of the proposed method, we conducted detailed experiments on three datasets, namely Market1501, MSMT17, and DukeMTMC-reID, as shown in Table 3. Compared with the baseline, our method improves mAP and Rank-1 by 2.9% and 1.1% on Market1501, 9.4% and 7.5% on MSMT17, and 5.2% and 3.5% on DukeMTMC-reID, respectively. These results demonstrate the strong generalization ability of the proposed method.

#### Effectiveness of MLCOR

The proposed MLCOR strategy aims to recover and utilize discriminative information from outliers to enhance model robustness. The confidence partition in MLCOR determines how outliers with different reliability levels are processed. Specifically, the confidence score is divided into four intervals by three boundaries, corresponding to outliers, low-confidence samples, medium-confidence samples, and high-confidence samples. These boundaries control the trade-off between recovering informative outliers and suppressing noisy pseudo-labels. A lower outlier boundary allows more samples to participate in refinement but may increase the risk of introducing incorrect pseudo-labels, whereas a higher boundary makes the recovery process more conservative and may leave useful boundary samples unused. The medium- and high-confidence boundaries further determine whether an outlier is processed by weighted neighbor voting or direct label inheritance. As shown in Table 4, we evaluated several representative confidence interval settings on Market1501. The adopted partition, with 0.00-0.40 for outliers, 0.40-0.60 for low-confidence samples, 0.60-0.80 for medium-confidence samples, and 0.80-1.00 for high-confidence samples, achieves stable and competitive performance. It should be noted that Table 4 provides a representative comparison of different confidence partitions rather than an exhaustive evaluation of all possible threshold combinations. In our experiments, this partition was selected on Market1501 and then kept unchanged on DukeMTMC-reID and MSMT17. Moreover, the ablation results on the three datasets show that MLCOR consistently improves the baseline performance, suggesting that the adopted confidence partition has reasonable stability and cross-dataset applicability under our experimental settings.

Furthermore, we employ a weighted neighbor voting strategy to handle low-confidence samples, refining and correcting pseudo-labels for outliers based on neighborhood label distribution. This strategy is particularly beneficial on more complex datasets. On MSMT17 and DukeMTMC-reID, mAP improvements of 1.1% and 1.8% are achieved, respectively, with Rank-1 improving by 1.6% on both datasets. On Market1501, the improvement is relatively modest, with mAP and Rank-1 increasing by 0.4% and 0.6%, respectively. These results suggest that the MLCOR strategy demonstrates better adaptability and effectiveness in complex and diverse scenarios, where pseudo-label noise and ambiguous boundary samples are more common. We further investigated two strategies for correcting labels of low-confidence outliers.

**Table 4:** We compare performance under different confidence interval settings. Bold represents the adopted confidence segmentation strategy.

Outlier	Low-confidence	Medium-confidence	High-confidence	mAP	R1
0.00-0.20	0.20-0.40	0.40-0.60	0.60-1.00	86.3	93.7
0.00-0.30	0.30-0.50	0.50-0.70	0.70-1.00	86.6	94.3
0.00-0.25	0.25-0.50	0.50-0.75	0.75-1.00	86.0	93.9
0.00-0.10	0.10-0.45	0.45-0.80	0.80-1.00	86.5	94.2
**0.00-0.40**	**0.40-0.60**	**0.60-0.80**	**0.80-1.00**	**86.8**	**94.4**

**Table 5:** We compare the performance of two methods on the DukeMTMC-reID dataset.

Method	mAP	ACC	NMI
Direct Neighbor	74.3	80.81	94.73
Weighted Neighbor	75.7	82.06	95.02

**Fig. 5:**
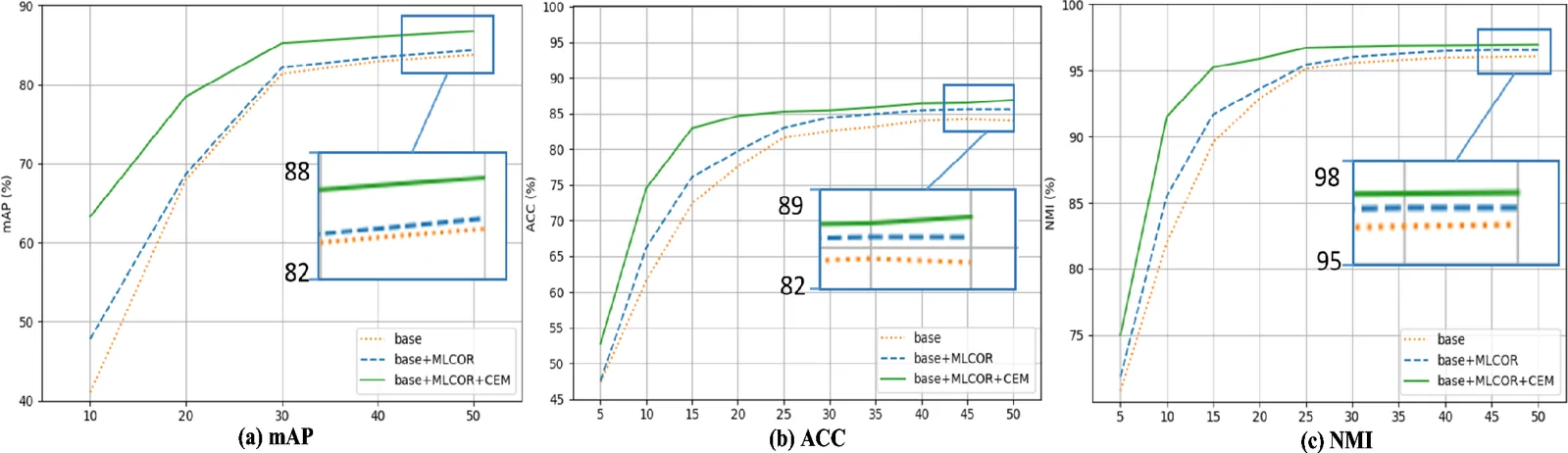
Evaluation of retrieval performance, classification performance, and clustering accuracy of our method using mAP, ACC and NMI scores on the Market1501 dataset.

The first method directly assigns the label of the nearest neighbor point; the second employs a weighted neighbor strategy that performs weighted computation of pseudo-labels based on neighborhood distribution and assigns pseudo-labels meeting score requirements to outliers. Detailed experiments conducted on the DukeMTMC-reID dataset show performance metric comparisons in Table 5, with data indicating that the weighted neighbor strategy is more effective for handling low-confidence points. Additionally, Fig. 5 presents comprehensive performance evaluation results for the proposed method, where mAP, ACC, and NMI are employed to measure retrieval capability, classification accuracy, and clustering consistency, respectively.

#### **Effectiveness of**$\boldsymbol{L_{C-DTL}^{zong}}$

The proposed loss function $L_{C-DTL}^{zong}$ not only provides constraints for local structures but also effectively extracts fine-grained information by introducing the similarity relationships between multi-level features and cluster centers, thus enhancing the model’s discriminative ability. Specifically, by calculating the distance between samples and each cluster center, the model is able to assess sample similarity across multiple scales. Through the incorporation of multi-level feature modeling, the Cluster-Driven Triplet Loss enables the model to consider multi-dimensional information of the samples comprehensively, allowing for effective discrimination at a fine-grained level. On MSMT17 and DukeMTMC-reID, our method improves mAP by 1.6% and 2.0%, respectively, compared to the baseline, and achieves a 0.7% improvement on Market1501. These results strongly demonstrate that combining multi-level features with cluster centers, along with the Cluster-Driven Triplet Loss, significantly enhances the model’s ability to extract fine-grained features and effectively constrains the local space, thus validating the effectiveness of the proposed method.

#### Effectiveness of $\boldsymbol{ L_{GEL}}$

The loss function $L_{GEL}$ proposed in this paper optimizes the embedding space structure by introducing a category-level similarity distribution. Specifically, $L_{GEL}$ enhances the compactness of same-class samples and the separability of different-class samples by considering the similarity between each sample and other samples in the global feature library, thereby creating a more discriminative boundary in the embedding space. As shown in Table 3, the performance shows significant improvement across all three datasets. Specifically, it achieves a 1.5% and 3.2% increase in mAP on Market1501 and DukeMTMC-reID, respectively, along with a 0.5% and 2.2% increase in Rank-1 accuracy. Notably, with $L_{GEL}$, the baseline achieves an 8.2% improvement in mAP and a 6.8% improvement in Rank-1 on MSMT17. These results demonstrate the crucial role of global feature optimization and sample similarity modeling through global embedding loss in enhancing discriminability and improving model performance, thereby validating the effectiveness of the global embedding loss.

**Fig. 6:**
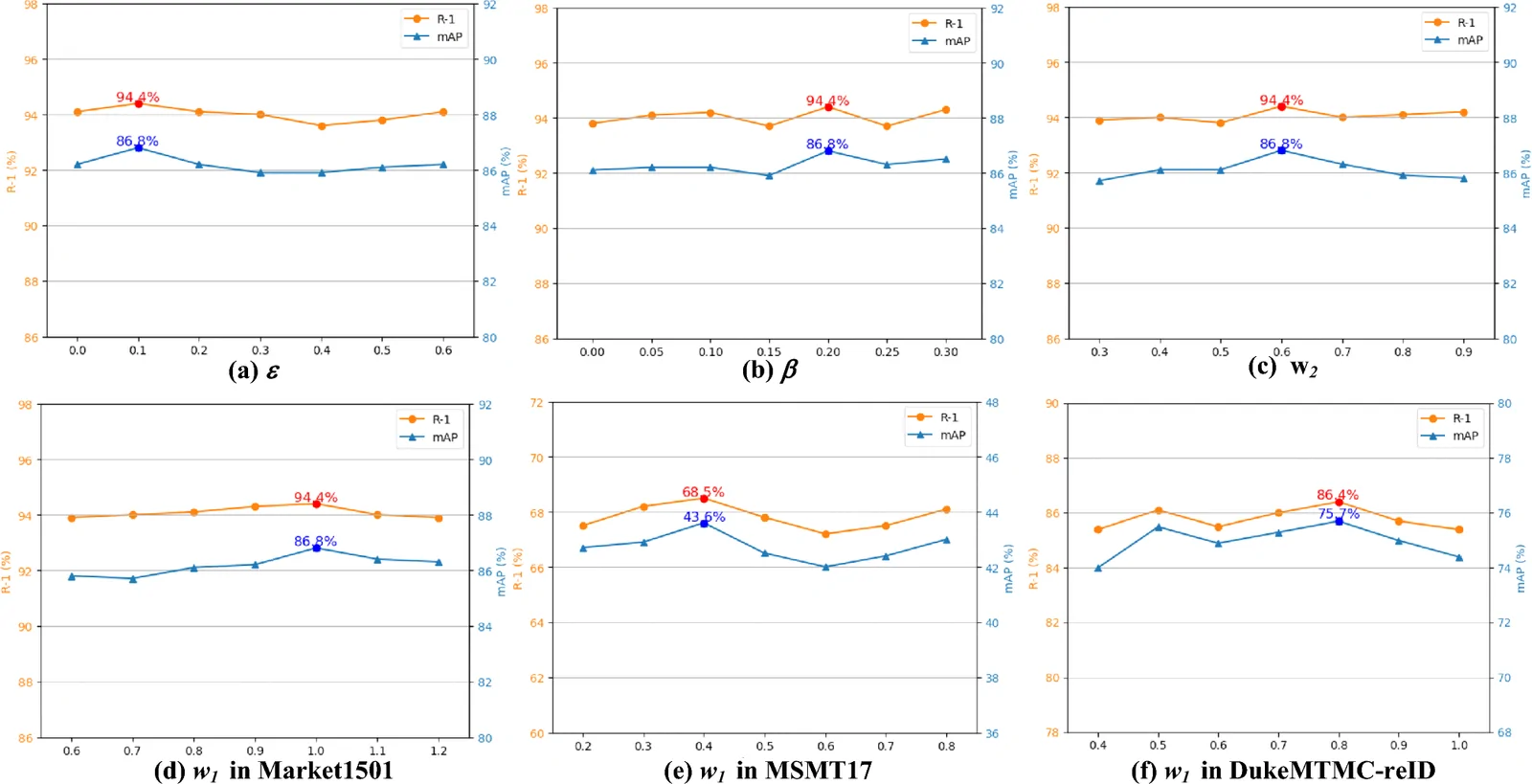
Parameter sensitivity analysis for different values of *\varepsilon*, *β*, and $w_2$ on Market1501. Note that $w_1$ is evaluated separately on three different datasets.

#### Analysis of *\varepsilon* and *β*

As shown in Fig. 6, we conducted a series of experiments on the dictionary update parameter *\varepsilon* using the Market1501 dataset. During the local dictionary update process, the centroids related to the input samples are updated, while the global dictionary only updates the entries corresponding to the sample indices, without affecting other positive samples. This ensures that each sample maintains independent discriminative features. Dictionary updates help the model dynamically adjust and optimize feature representations, thereby improving recognition accuracy. Keeping other hyperparameters unchanged, we found that the optimal value is 0.1, which also indicates that more emphasis is placed on new features during the feature update process. Additionally, we used *β* to adjust the contribution of the local loss in Eq. (25). The upper and lower-level features are derived from local views, and the hyperparameter *β* controls the relative importance of local features in the loss calculation. By tuning this value, the model can flexibly strike a balance between global and local features. Keeping other hyperparameters constant, by comparing the model performance under different weight settings, we found that setting the weight of the local features to 0.2 resulted in the optimal performance improvement. Increasing the weight of the local features can enhance attention to finer details, but may suppress the expression of global features, thus affecting overall recognition performance. Conversely, reducing the weight of local features leads to insufficient attention to them. Setting the weight to 0.2 ensures that global features dominate the re-identification task, while local features can still contribute their unique information. This balance enables the model to exhibit good recognition capabilities across different scenarios.

#### Analysis of $w_1$ and $w_2$

As shown in Fig. 6, we further analyze the effects of $w_1$ and $w_2$ on model performance. In Eq. (28), $w_1$ and $w_2$ control the contributions of $L_{C-DTL}^{zong}$ and $L_{GEL}$ to the final loss, respectively. The coefficient $w_1$ regulates the strength of the local structural constraints introduced by $L_{C-DTL}^{zong}$. Since local neighborhood structures may vary across datasets due to differences in sample distribution, background complexity, and pseudo-label noise, the influence of $w_1$ is further analyzed on different datasets. As shown in Fig. 6d–f, the sensitivity of $w_1$ is evaluated on Market1501, MSMT17, and DukeMTMC-reID, respectively. The results show that the suitable value of $w_1$ tends to decrease as the dataset complexity increases. Specifically, $w_1$ is set to 1.0, 0.8, and 0.4 for Market1501, DukeMTMC-reID, and MSMT17, respectively. This observation indicates that a relatively large $w_1$ is beneficial when the local neighborhood structure is reliable, while a smaller $w_1$ is more suitable for complex datasets with noisier pseudo-labels and less reliable local structures. Therefore, the selection of $w_1$ reflects the trade-off between exploiting local fine-grained relationships and suppressing unreliable local constraints. In contrast, $L_{GEL}$ optimizes the discriminative ability of the embedding space from a global perspective and shows stronger consistency across different datasets. Therefore, $w_2$ is fixed to 0.6 in all experiments. A fully adaptive selection strategy for $w_1$ based on pseudo-label reliability or local neighborhood reliability will be explored in future work.

**Fig. 7:**
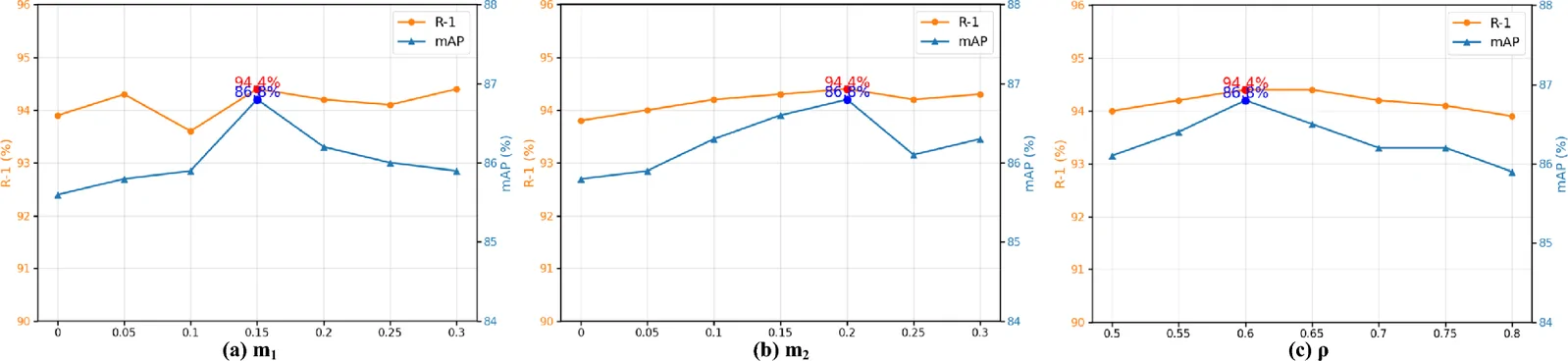
Parameter sensitivity analysis for different values of $m_1$, $m_2$, and *ρ* on Market1501.

#### Analysis of $m_1$, $m_2$, and *ρ*

To further evaluate the robustness of the proposed method, we conduct sensitivity analyses on three key hyperparameters, namely $m_1$, $m_2$, and *ρ*, as shown in Fig. 7. The parameter $m_1$ controls the fusion ratio between global and local distance matrices. The results show that the performance first improves and then slightly declines as $m_1$ increases, with the best performance achieved around $m_1 = 0.15$. When $m_1$ is too small, the model mainly relies on global features, which weakens the contribution of local discriminative cues. Conversely, when $m_1$ is too large, the model overemphasizes local features, which may introduce noise and reduce the reliability of similarity estimation. Therefore, a moderate value of $m_1$ achieves a good balance between global consistency and local discrimination. The parameter $m_2$ balances the contributions of multi-level cross-entropy losses. When $m_2 = 0$, the model degenerates to using only global supervision. As $m_2$ increases, the incorporation of local branch supervision improves the discriminative capability and leads to better performance. However, when $m_2$ becomes too large, the local branches dominate the optimization process and may interfere with global representation learning. The optimal performance is achieved around $m_2 = 0.2$, indicating a proper balance between global and local supervision. The parameter *ρ* determines whether low-confidence samples are accepted during the outlier refinement process. The results indicate that the performance reaches the optimum around *ρ = 0.6*. A smaller *ρ* allows more low-confidence samples to be selected, which increases the risk of introducing noisy labels. In contrast, a larger *ρ* makes the selection overly conservative, resulting in insufficient utilization of useful samples. Therefore, *ρ* plays a crucial role in balancing sample recovery and noise suppression. Overall, the performance varies smoothly across a reasonable range of values, demonstrating that the proposed method is robust to parameter variations and does not rely on exhaustive manual tuning. Other mechanism-related parameters (e.g., *σ*, *s*, and *B*) are set to empirically effective values. Specifically, *σ* and *B* are fixed across datasets, while *s* is adjusted within a small range according to the data scale and neighborhood density of each dataset. In practice, the performance remains stable under these settings.

**Fig. 8:**
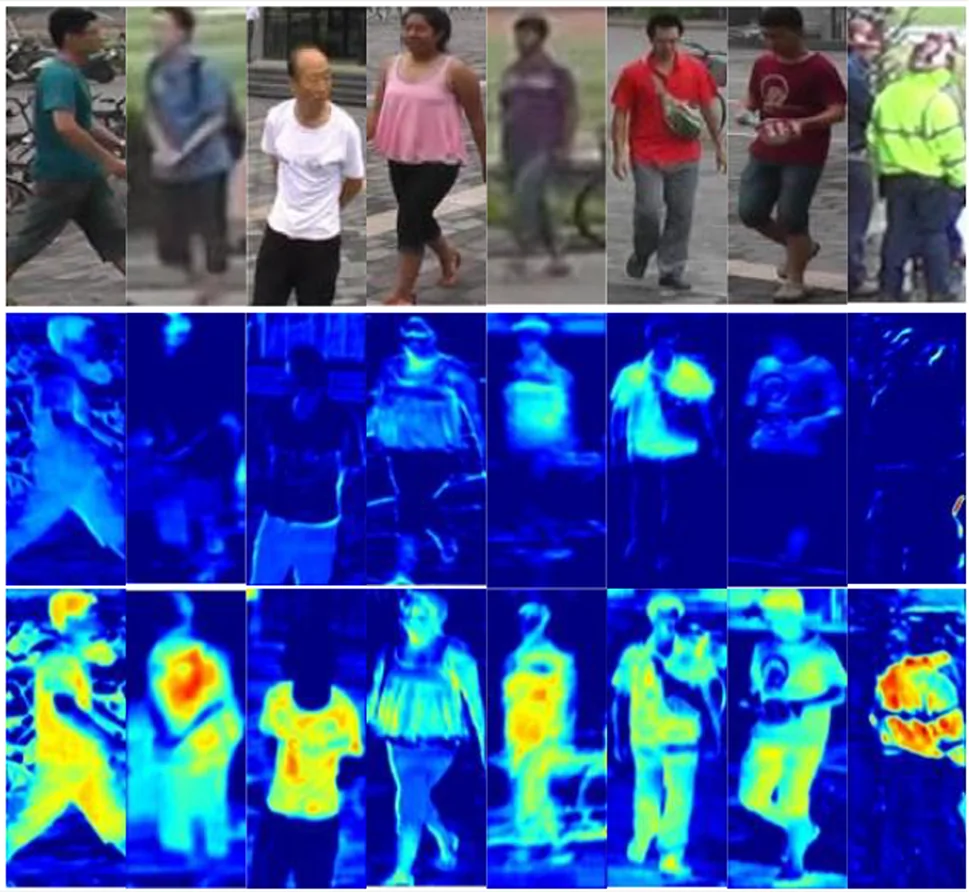
Qualitative comparison between the baseline and our method on randomly selected samples from the training set of Market1501. The first row shows the original images, the second and third rows show the Grad-CAM heatmaps generated by the baseline and our method, respectively.

### Visualization

**Fig. 9:**
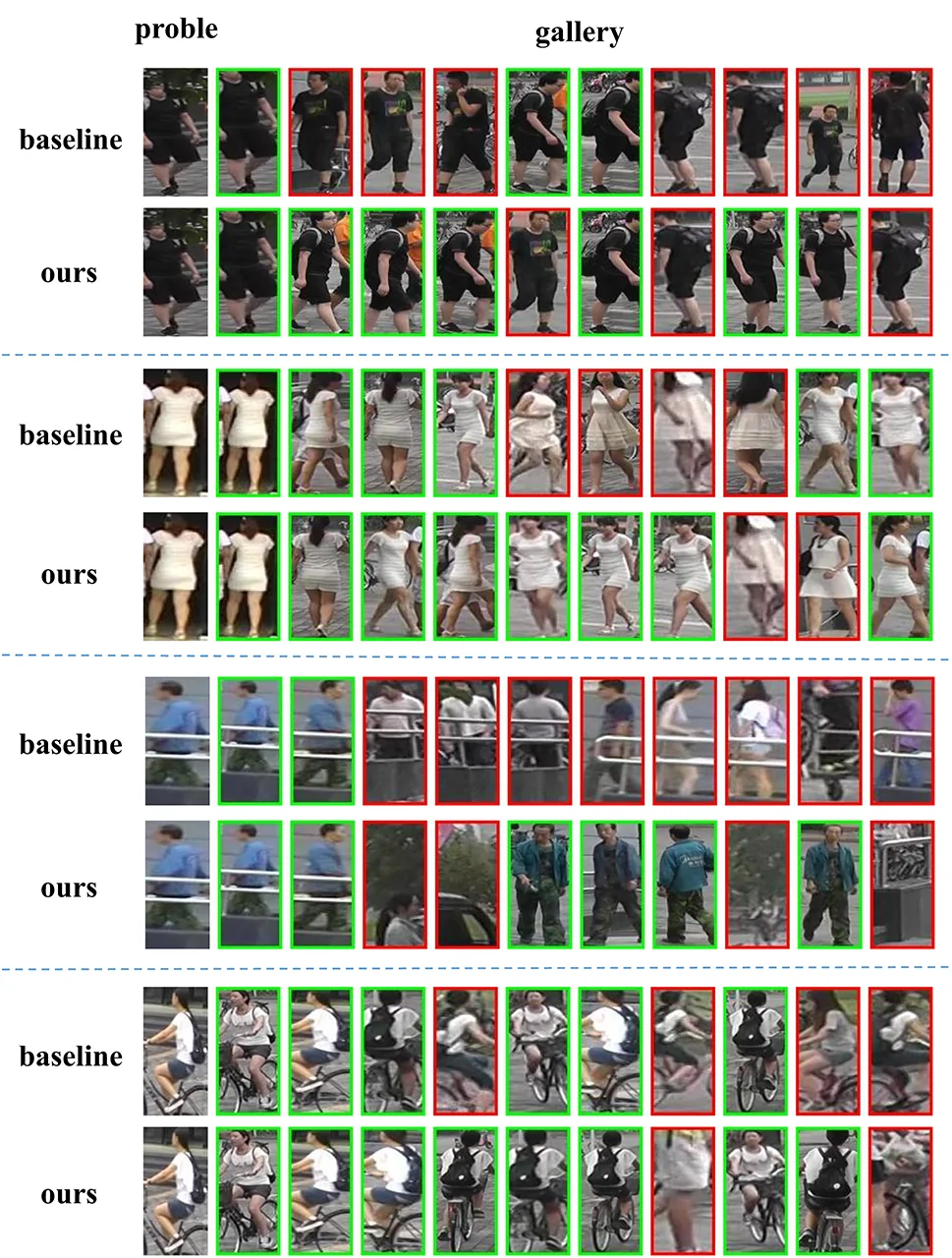
Qualitative analysis on the baseline and our model (top 10 retrieval results on the Market1501 dataset). The green box indicates a correct retrieval, while the red box indicates an incorrect retrieval.

To further validate our approach, we conducted a visualization experiment on the training set of the Market1501 dataset. In Fig. 8, we present Grad-CAM heatmap visualizations for eight representative individuals. From the heatmaps, it can be observed that the model exhibits strong responses not only in semantically important regions, but also around object boundaries. This suggests that the proposed method is able to capture more discriminative structural cues, which is beneficial for fine-grained person representation learning. In addition to the heatmaps, we also performed a retrieval visualization. Representative retrieval results are shown in Fig. 9, where each row includes a query image and its top 10 results. The first image on the left in each row represents the query image, and the other images correspond to the retrieval results. Green boxes indicate correct retrievals, while red boxes indicate incorrect retrievals. By comparing the baseline retrieval results with ours, it is clear that our method demonstrates superior performance. This approach is more sensitive to fine-grained details, with its embedding space exhibiting higher discriminability, resulting in improved recognition accuracy.

## Conclusion

This paper proposes an optimization solution that combines Multi-level Confidence Outlier Refinement and Collaborative Embedding Method, aiming to enhance the representational capacity of the embedding space and improve the accuracy of pseudo-labels. By introducing the outlier refinement mechanism, we can accurately recover outliers, effectively enriching feature representations while reducing noise interference, thus improving the quality and reliability of pseudo-labels. In addition, this paper implements the complementarity between global semantic information and local structure through the collaborative embedding method, enhancing the discriminative capability of the embedding space. By integrating multi-level similarity relationships, the sensitivity to fine-grained features is improved, and by focusing on the hardest negative samples, the clarity of inter-class boundaries is further enhanced. Comprehensive experiments demonstrate significant improvements in clustering accuracy and pseudo-label accuracy, achieving excellent performance across three commonly used person re-identification datasets, with strong accuracy and robustness in complex environments. In the future, we will further study the outlier refinement and label assignment strategies to promote their practical deployment and application.

## Data Availability

The datasets used and analyzed during the current study are available from the corresponding author on reasonable request.

## Footnotes

1 The DukeMTMC benchmark dataset and its derivatives, including DukeMTMC-ReID, have been requested to be removed from public online access until further notice.
